# Exhaustion of T cells after renal transplantation

**DOI:** 10.3389/fimmu.2024.1418238

**Published:** 2024-08-06

**Authors:** Xiujia Wang, Jinghui Zhang, Pingshan Zhong, Xiuwang Wei

**Affiliations:** ^1^ Department of 1st Urology Surgery, The People’s Hospital of Guangxi Zhuang Autonomous Region, Nanning, China; ^2^ Department of Surgery and Oncology, Graduate School of Medical Sciences, Kyushu University, Fukuoka, Japan

**Keywords:** T cell exhaustion, renal transplantation, allograft rejection, graft survival, immunotherapy

## Abstract

Renal transplantation is a life-saving treatment for patients with end-stage renal disease. However, the challenge of transplant rejection and the complications associated with immunosuppressants necessitates a deeper understanding of the underlying immune mechanisms. T cell exhaustion, a state characterized by impaired effector functions and sustained expression of inhibitory receptors, plays a dual role in renal transplantation. While moderate T cell exhaustion can aid in graft acceptance by regulating alloreactive T cell responses, excessive exhaustion may impair the recipient’s ability to control viral infections and tumors, posing significant health risks. Moreover, drugs targeting T cell exhaustion to promote graft tolerance and using immune checkpoint inhibitors for cancer treatment in transplant recipients are areas deserving of further attention and research. This review aims to provide a comprehensive understanding of the changes in T cell exhaustion levels after renal transplantation and their implications for graft survival and patient outcomes. We discuss the molecular mechanisms underlying T cell exhaustion, the role of specific exhaustion markers, the potential impact of immunosuppressive therapies, and the pharmaceutical intervention on T cell exhaustion levels. Additionally, we demonstrate the potential to modulate T cell exhaustion favorably, enhancing graft survival. Future research should focus on the distinctions of T cell exhaustion across different immune states and subsets, as well as the interactions between exhausted T cells and other immune cells. Understanding these dynamics is crucial for optimizing transplant outcomes and ensuring long-term graft survival while maintaining immune competence.

## Introduction

Renal transplantation is the preferred treatment option for end-stage renal disease (ESRD), offering improved quality of life and survival compared to dialysis (1). However, rejection remains a formidable challenge to the long-term success of renal transplants, compounded by the complications arising from the use of immunosuppressants, such as infections and tumors (2). Despite the immunosuppression, T cells remain the central reactive entity against infections and tumors, underscoring the importance of investigating the functional status of T cells in this context (3). Rejection episodes in kidney transplants are characterized by the infiltration of immune cells into the kidney, with two main types being identified: T cell-mediated rejection (TCMR) and antibody-mediated rejection (ABMR) (4). T cells can recognize alloantigens via two distinct pathways: the direct pathway, where T cells recognize intact allogeneic major histocompatibility complex (MHC) molecules on donor cells, and the indirect pathway, where T cells recognize donor MHC peptides processed and presented by recipient antigen-presenting cells (APCs) (5). Chronic rejection is hypothesized to occur after donor dendritic cells (DCs) are replaced by recipient DCs within the allograft, primarily through the indirect pathway (6). During chronic rejection, although direct killing of the graft by T cells is rare, T helper cells and regulatory T cells can contribute to graft damage and immune conditions by secreting cytokines and soluble mediators (7).

T cell exhaustion arises during persistent antigen exposure, characterized by the progressive loss of effector functions (diminished proliferation, cytokine production, and cytotoxic capabilities), sustained expression of inhibitory receptors, dysregulation of metabolism, and a distinct transcriptional state (8). This phenomenon was first identified in CD8+T cells from murine models chronically infected with lymphocytic choriomeningitis virus (9) and has since been extensively studied across various chronic infections (10, 11), tumors (12–14), and autoimmune diseases (15, 16). Under different circumstances, T cell exhaustion is intricately regulated by various networks of inhibitory molecules, transcription factors, and signaling pathways, leading to varying outcomes (17). In renal transplantation, T cell exhaustion has emerged as a potential regulatory mechanism for modulating alloreactive T cell responses, thereby fostering graft acceptance (18, 19). This delicate balance highlights the need for targeted interventions that can modulate the immune response precisely, offering a pathway to improved transplant success rates and recipient health. Developing therapeutic approaches that can selectively induce exhaustion in alloreactive T cells, while preserving robust anti-tumor and anti-pathogen responses, is poised to be a pivotal strategy in enhancing outcomes for organ transplant recipients. Therefore, a nuanced comprehension of the molecular underpinnings of T cell exhaustion after renal transplantation is imperative.

## Molecular mechanisms of T cell exhaustion

T cell exhaustion is distinguished from other forms of lymphocyte dysfunction by its unique molecular signatures and surface phenotypes (20). Unlike an “all or none” phenomenon, T cell exhaustion unfolds as a gradual, hierarchical process, typically requiring weeks or months of sustained antigen stimulation to fully manifest (21, 22). This complex phenomenon involves a diverse array of inhibitory receptors, transcription factors, and signaling pathways, which can vary across different conditions and even within different phases of the same disease (19, 23). For example, low oxygen and high lactate levels in the tumor microenvironment (TME) can promote T cell exhaustion by modulating the eATP–adenosine axis (24). In the context of transplantation, immunosuppressants have become a prominent factor affecting T cell exhaustion. Understanding the molecular mechanisms underlying T cell exhaustion after renal transplantation is crucial and could significantly benefit transplantation recipients.

Programmed cell Death 1 (PD-1), a transmembrane receptor that belongs to the CD28/cytotoxic T-lymphocyte associated protein 4 (CTLA-4) family, acts as a costimulatory signal inhibitor for T cell receptor (TCR) activation (25). The production of PD-1 is upregulated via TCR recognition of MHC, thereby preventing the overactivation of T cells and limiting immune-mediated damage to native tissue (26). Studies have explored the dynamics of PD-1 expression and its association with T cell exhaustion in the context of renal transplantation, shedding light on its implications for transplant outcomes. Yucheng et al. (27) conducted flow cytometry analysis on whole blood samples from kidney transplantation recipients and found that PD1+CD57- marked exhausted T cells were elevated in recipients with stable renal function but decreased in those experiencing acute rejection. Thiago et al. studied the effects of PD-1’s presence in mice, and showed that overexpression of PD-1 on T cells promotes allograft tolerance in a fully MHC-mismatched cardiac transplant model (28). Modulating PD-1 signaling could be a strategic approach to enhancing transplant tolerance while maintaining immune competence against infections and malignancies.

T-cell immunoglobulin and mucin containing protein-3 (TIM-3) plays a nuanced role in the immune response, modulating the function of CD4+CD25+ regulatory T cells, inhibiting aggressive Th1 cells mediated auto- and allo-immune responses, and promoting T cell exhaustion (29). Engagement of TIM3 on T cells and DCs provides different tyrosine phosphorylation patterns, lead to varied effects (30). Early studies found that TIM-3 mRNA was highly expressed in graft and urinary samples from acute rejection patients compared to stable transplants (31, 32). Not only in the renal graft but a peripheral CD4 T cell-exhausted phenotype, characterized by increased expression of PD-1 and TIM-3, was also associated with renal graft survival (33). Moreover, T cell exhaustion is not only related to graft survival but also affects recovery from infections after transplantation. A study highlighted that the absence of PD-1 and TIM-3 exhaustion markers on BK virus-specific T cells correlated with shorter clearance times for the virus in renal transplantation patients (34). Further study targeting TIM-3 may serve as a promising strategy to prevent chronic allograft rejection and promote tolerance.

T cell immunoreceptor with immunoglobulin and ITIM domain (TIGIT) plays a significant role in inhibiting T cell activation, proliferation, and the acquisition of effector functions. The TIGIT/CD226 axis represents a newly identified pathway that is critical for regulating T cell activity (35). This axis includes TIGIT, a co-inhibitory receptor along with CD226, which performs a co-stimulatory function and shares ligands with TIGIT (36). Arnaud et al. discovered a correlation between allospecific T cell hyporesponsiveness and increased expression of TIGIT post-transplantation, implicating the CD226/TIGIT axis in this process (37). Amy et al. identified TIGIT as a marker for the polyfunctional donor-reactive CD4+ T cell population, whose decline following kidney transplantation may contribute to allograft tolerance (38). These findings suggest that elevated TIGIT expression could indicate a mechanism through which the immune system modulates responses to the transplanted organ, potentially contributing to tolerance and graft survival.

Lymphocyte-activation gene 3 (LAG-3), characterized by a structural resemblance to CD4, facilitates inhibitory function through selective interaction with stable complexes of peptides and MHC class II (39). LAG-3 is highly expressed, particularly on CD8+ T cells exhibiting an exhausted phenotype in the context of chronic viral infections and cancer (40, 41). While LAG-3 has been less directly highlighted in renal transplantation, its co-expression with PD-1 has been identified as a potent enhancer of T cell exhaustion, contributing to a decreased likelihood of rejection (42).

CTLA-4, a crucial co-inhibitory molecule rather than a co-stimulatory one, competes with CD28 for binding to their shared B7 ligands (CD80/CD86) on APCs. Thereby interfering with TCR-mediated signal transduction (43). Given CTLA-4’s key role in regulating allograft rejection and tolerance (44), significant attention has been focused on the relationship between CTLA-4 genetic variations and graft outcomes following solid organ transplantation.

Studies on T cell exhaustion after transplantation, based on characteristic molecules, have shown that exhausted T cells exist not only in the renal grafts but also in peripheral tissues and the circulatory system. This presence affects both graft survival and the overall immune function of post-transplant patients. Further research and understanding of the molecular mechanisms of T cell exhaustion could help improve outcomes for renal transplantation recipients.

## Transcription factors variation of T cell exhaustion

Transcription factors such as the nuclear factor of activated T cells (NFAT), basic leucine zipper ATF-like transcription factor (BATF), and thymocyte selection-associated high-mobility group box protein (TOX), play crucial roles in regulating the expression of inhibitory receptors and the exhaustion program (45, 46). NFAT, in particular, has been implicated in various T cell states, including activation (47), exhaustion (48), tolerance (49), and anergy (50). The context of TCR engagement—whether there’s persistent antigen stimulation in the absence of positive co-stimulation or in the presence of negative costimulatory signals—becomes crucial for determining T cell fate (51, 52).

*In vivo* studies have revealed that NFAT directly influences CD8+ T cell exhaustion by binding to regulatory regions of genes associated with exhaustion, such as the promoters of PDCD1 (PD-1) and HAVCR2 (TIM-3) (45). Transcriptional profiling has indicated elevated expression of NFATc1 (NFAT2) in exhausted CD8+ T cells with chronic viral infection (53). Suggesting that increased transcription of Nfatc1 could correlate with inadequate activation or translocation of this transcription factor during T cell exhaustion. The role of NFAT is particularly relevant in the context of kidney transplantation, where patients often receive immunosuppressive treatments including Calcineurin Inhibitors (CNIs). CNIs target the NFAT phosphorylation process, thereby reducing IL-2-mediated T lymphocyte activation, proliferation (54) and cytotoxicity (55). Interestingly, studies involving allogeneic Hematopoietic Stem Cell Transplantation (HCT) patients (56) and Chimeric antigen receptor (CAR) T cells (57) have shown that CNI treatment can inhibit the terminal differentiation of donor exhausted T cells post-transplantation.

**Figure 1:**
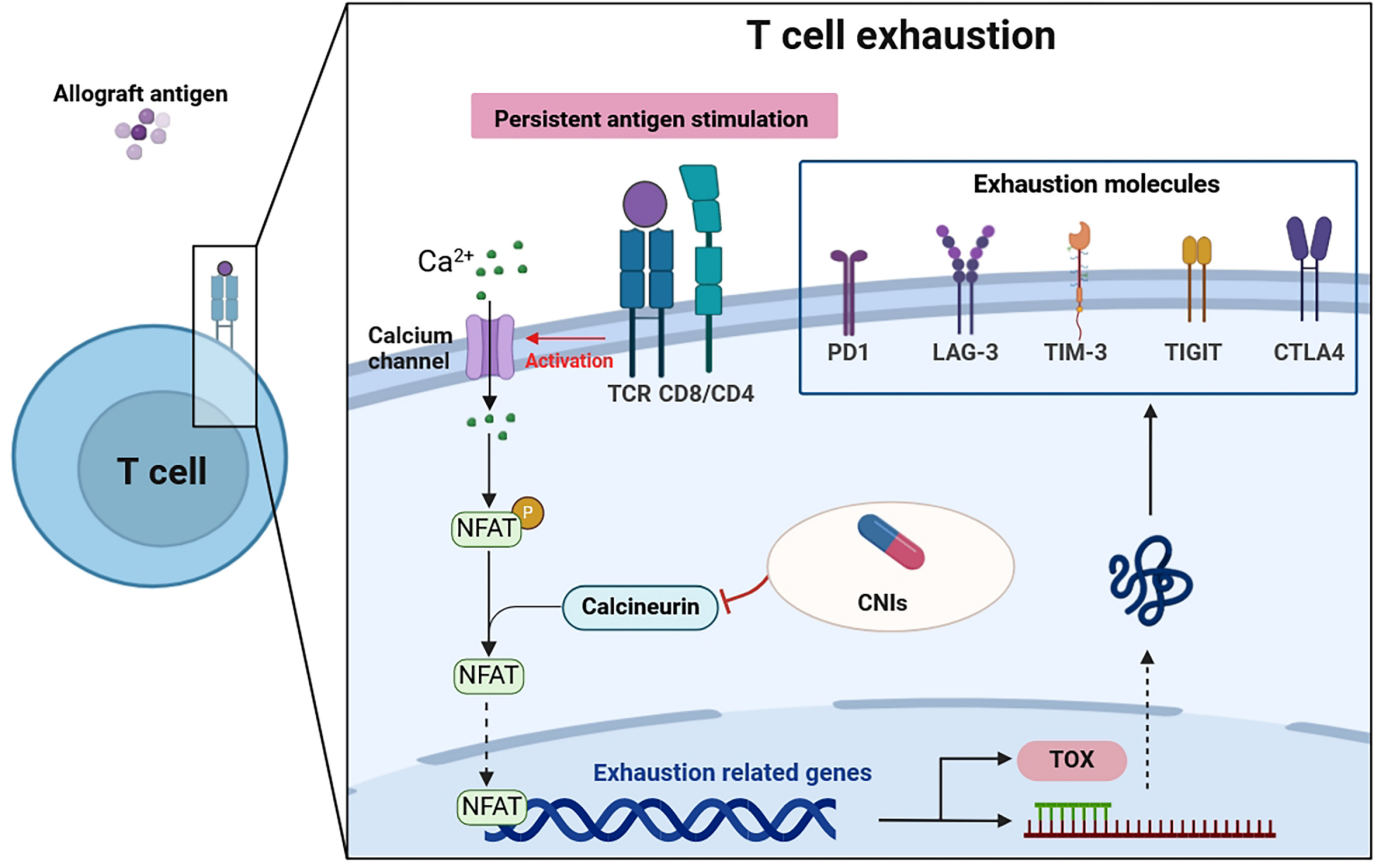
T cell exhaustion in patients after renal transplantation. Persistent antigen stimulation of the TCR activates the calcineurin pathway. Calcineurin dephosphorylates cytoplasmic NFAT, which then moves into the nucleus to regulate genes that govern either T cell effector function or T cell exhaustion. CNIs, used in transplantation recipients, can inhibit this process. Furthermore, NFAT induces the transcription factors such as TOX, which drive the exhaustion program, expressing exhaustion molecules. Thus, the transmission of extracellular signals by NFAT is a critical step in T cell exhaustion. TCR, T cell receptor; CNIs, calcineurin inhibitors; PD1, programmed death-1; LAG-3, lymphocyte-activation gene 3; TIM-3, T-cell immunoglobulin-3; TIGIT, T-cell immunoglobulin and ITIM domain; CTLA4, cytolytic T lymphocyte-associated antigen 4; NFAT, nuclear factor of activated T cells; TOX, thymocyte selection-associated high-mobility group box protein.

Recent research has illuminated the pivotal role of the TOX in T cell biology, revealing that while effector and memory T cells can develop in the absence of TOX, exhausted T cells cannot (58, 59). This differentiation underscores TOX’s unique role in T cell exhaustion. TOX essentially translates the early and sustained activity of NFAT2 (a form of NFAT) into a later, calcineurin-independent, TOX-driven molecular and epigenetic program characteristic of exhausted T cells (60). In doing so, TOX not only suppresses terminal effector T cell-specific epigenetic events but also initiates critical exhausted T cell-specific epigenetic modifications (61, 62). These findings position TOX as an essential transcriptional and epigenetic orchestrator of the T cell exhaustion program. In the context of renal transplantation, the calcineurin-NFAT2 pathway emerges as a critical initiator required to induce TOX expression. Consequently, the use of CNIs in transplant recipients may inadvertently impede the transcriptional and epigenetic frameworks essential for fostering T cell exhaustion (58, 63). Given that T cell exhaustion plays a role in modulating immune responses to the transplant, preventing graft rejection, and maintaining tolerance, the impact of CNIs on this process is of particular significance (**Figure 1**).

## T cell exhaustion and graft survival

The dynamics between T cell exhaustion and the long-term survival of renal transplants are complex and multifaceted, making it a focal point of ongoing research. On one hand, evidence suggests that repeated exposure to alloantigens leads to the gradual exhaustion of T cells, potentially aiding in the acceptance of the graft (64–66). Complementing these findings, Miguel et al. revealed that the proportion of exhausted T cells six months post-transplant was positively associated with the estimated glomerular filtration rate (eGFR) at the same timeframe of 26 renal transplantation recipients (67). Through the male-to-female skin transplant model, researchers found that both CD8+ and CD4+ T cells exhibited exhaustion signatures positively correlated with alloantigen load, promoting transplant acceptance (68). This observation may help explain why organs with high antigen loads, such as the liver, tend to be better tolerated by the recipient’s immune system compared to smaller organs. On the other hand, excessive exhaustion might compromise the immune system’s capacity to fend off viral infections and combat tumors, potentially heightening the risk of adverse outcomes and graft rejection (69). Further emphasizing the double-edged sword of T cell exhaustion, Mysore et al. presented a longitudinal study of liver transplant patients, establishing a strong link between exhausted T cells and the occurrence of infectious episodes (70). Collectively, these studies illuminate the delicate balance in transplant immunology: T cell exhaustion can facilitate transplant acceptance but may also diminish the immune system’s efficacy against chronic infections and tumors, presenting a challenge in managing transplant patient health optimally.

However, there is much to discuss regarding the protection of grafts by exhausted T cells. Studies using mouse models for kidney and islet transplants have shown that tissue-resident memory (TRM) cells migrate to the transplant site and engage in chronic rejection processes, with only a small proportion exhibiting signs of exhaustion (71, 72). This suggests that while T cell exhaustion may play a role in promoting graft tolerance, the presence and activity of non-exhausted TRM cells could counteract this effect by contributing to the processes leading to chronic rejection. Masahiro etal (73), conducted a study on acute graft-versus-host disease (aGVHD) in murine models, focusing on direct renal damage mediated by allogeneic donor T cells. They found that donor MHC+ T cells, encompassing both CD4+ and CD8+ subsets, exhibited heightened activation and exhaustion markers, alongside increased secretion of pro-inflammatory cytokines and cytotoxic proteins, contributing to injury in renal endothelial and tubular epithelial cells. Notably, despite the elevated exhaustion markers, these T cells retained their cytotoxic capabilities, underscoring that the presence of inhibitory receptors does not necessarily equate to functional exhaustion. This distinction is crucial in research to accurately identify the state of T cell exhaustion.

A notable exception to the typical effects of T cell exhaustion involves CD4+FOXP3+ regulatory T cells (Tregs). Research indicates that conditions of chronic immune activation, whether due to autoimmune diseases or persistent infections, can lead to an accumulation of PD-1-expressing Tregs that exhibit diminished functional activity (74, 75). Tan et al. identified reduced signaling through the PI3K–AKT pathway as a mechanism underlying the enhanced suppressive capacity of PD-1–deficient Treg cells (76). This phenomenon of potential Treg exhaustion could paradoxically increase the risk of harmful T cell responses against the transplanted kidney. Instead of promoting tolerance and protecting the graft, exhausted Tregs may lose their regulatory capacity, thus failing to prevent damaging immune responses that could compromise transplant survival (27, 77). This highlights the complexity of immune regulation in transplantation, where not only the quantity but the quality and functional state of regulatory cells are crucial for maintaining the balance between acceptance and rejection of the graft.

## Immune exhaustion

Although exhaustion has most commonly been studied in the context of CD8 T cell responses, recent studies indicate that chronic antigen exposure may also affect B cells, NK cells, and CD4 T cells in a parallel manner. For instance, exhausted NK cell have been associated with the expression of TIGIT but not CTLA-4 and PD-1 in tumor patients (78). In the study of malaria, exhausted CD4 T cell was found to exhibit reduced T-bet expression and mTORc1 activity (79). An exhausted phenotype of Th2 cells was recognized after allergen-specific immunotherapy, the elevated exhaustion markers (PD-1, CTLA-4) enhanced Th2 response and even exacerbated allergic airway inflammation (80). Moreover, in the immune microenvironment, exhausted T cells also interact with other cells. For example, T follicular helper cells (Tfh) are essential to sustain functions of exhausted T cells. Studies of tumor microenvironment showed that exhausted T cells recruit Tfh, through CXCL13 and BLIMP1/TCF1 axis, and regain cytotoxicity (81, 82). These studies highlight that research on immune exhaustion in transplant patients is far less developed than research on tumor infection and autoimmunity. A deeper understanding of the depletion of different immune cells and their roles in the context of exhausted effector T cells can help transplant patients better control graft rejection and fight against tumors and infections.

## Therapeutic modulation of T cell exhaustion

To enhance graft survival, pharmaceutical developments are focusing on drugs that target key molecules involved in T cell exhaustion. Belatacept, a high-affinity variant of CTLA4-Ig represents a significant advancement in this area and has been approved for kidney transplant recipients (83). Belatacept inhibits T lymphocyte proliferation and the production of cytokines such as interleukin-2, interferon-γ, interleukin-4, and TNF-α by binding to CD80 and CD86 on antigen-presenting cells, thereby blocking CD28-mediated costimulation of T cells (84). A long-term study reported the outcome of belatacept in renal transplantation, seven years after transplantation, patient and graft survival and the mean eGFR were significantly higher with belatacept than with cyclosporine (85). The use of belatacept exemplifies the ongoing efforts to modulate the molecule of T cell exhaustion post-transplant in a manner that preserves the graft while minimizing adverse outcomes. However, Budde and colleagues (86) highlighted the clinical implications of belatacept over a two-year follow-up period, showing that 5.4% of patients treated with CNIs developed cancer, compared to 8.1% in the belatacept group. This difference sheds light on the complex trade-offs involved in immunosuppressive therapy, balancing the reduction of rejection risks against potential side effects, including an increased risk of developing cancer.

The interplay between immunosuppressive drugs used in renal transplantation and immune checkpoint inhibitor (ICI) therapy for cancer creates a complex scenario regarding T cell exhaustion in the tumor microenvironment post-transplant. The rate of rejection after ICIs is highest among renal transplantation compared to liver, heart, and lung transplant patients and ranges from 41 to 48% (87). Sandhya et al. (88) emphasized the urgent need for awareness regarding the heightened risk of acute allograft rejection/failure following ICI therapy in renal transplant patients. In this systemic review, 40.9% (18/44) of patients were reported to have acute rejection with the median time from ICI initiation to acute rejection diagnosis of 24 days. A multi-center study by Murakami et al. (89) showed that, although there were potential improvements in cancer outcomes, ICIs were associated with a higher risk of rejection in kidney transplant recipients, with a 42% acute rejection rate in ICI-treated patients compared to 5.4% in non-ICI patients. Conversely, Robert and colleagues found no irretrievable allograft rejection without evidence of tumor response in 17 renal transplantation tumor patients (90). The CONTRAC-1 study also showed encouraging results concerning the use of anti-PD-1 for advanced cutaneous squamous cell carcinoma in renal transplant recipients, with an overall response rate of 46% (5/11) and no allograft rejection occurring (91).

The use of ICIs in renal transplant recipients poses a significant risk of allograft rejection, necessitating vigilant monitoring. Maintaining baseline immunosuppression before treatment with an immune checkpoint inhibitor in kidney transplant recipients might not affect expected efficacy and might reduce the risk of allograft rejection mediated by ICIs. Traditionally, T cell dysfunction within tumors and protein expression levels of immune checkpoints like PD-1 and CTLA-4 have been predictive markers for the efficacy of ICI therapy (92, 93). Barsch et al. (94) advanced the understanding within the hepatocellular carcinoma context, finding that high levels of exhausted T cells with increased expression of PD-1, LAG-3, and CTLA-4 negatively influenced patient prognoses, whereas memory T cells, expressing fewer immune checkpoints, correlated with better survival outcomes. Further complicating this landscape, Garnett et al. (95) observed in melanoma patients post-renal transplantation receiving ICI therapy, an expansion of alloreactive CD8+ T cells. This expansion, induced by ICI therapy, overlapped with cases of ICI-associated organ rejection, highlighting the delicate balance between treating cancer and maintaining graft survival. This complex interaction underscores the need for large-scale prospective studies to identify optimal immunosuppressive strategies that can both mitigate rejection risks and enhance cancer treatment outcomes, ensuring the well-being of transplant recipients undergoing cancer therapy (**Table 1**).

**Table 1:** T cells in tumor, transplantation and after ICIs.

Celltype	Original		ICIs	
	Transplantation	Tumor	Transplantation	Tumor
**Memory T cells** (CD103, ICOS, PD-1, TIGIT, and TIM-3)	-Chronic rejection	-Correlate with better oncological outcomes	-Responsible for acute rejection	-As effectors of immune checkpoint inhibitors
		-Dysfunction contributes to tumor development		-Spectrum of inflammatory toxicities
**Exhausted T cells** (PD-1, CTLA-4, CD39, and LAG-3)	-Graft survival	-Correlate with poor overall survival	-Poor responses to ICIs	
**Regulatory T cells** (FOXP3, CTLA-4, CD4)	-Allograft tolerance	-Promote tumor cell evasion		-Cell number reduced, yet suppressive function is maintained
		-Correlate with poor prognoses		-Mediates resistance and response to ICIs;

## Conclusion

T cell exhaustion occupies a nuanced position in the context of renal transplantation, presenting both advantageous and detrimental implications. Moderate levels of exhaustion may contribute to the regulation of alloreactive T-cell responses and promote graft acceptance, while excessive exhaustion may compromise the ability to control viral infections and tumors, posing potential risks to transplant recipients. The expression patterns of exhaustion markers and the impact of immunosuppressive therapies on T cell exhaustion levels warrant further investigation.

In recent years, the application of technologies such as implantable artificial kidneys (96), xenotransplantation of kidneys (97), and renal regeneration using bioengineered organoids (98) has become increasingly widespread in experimental settings. As we continue to explore these non-traditional kidney transplantation approaches, there is a growing need to pay closer attention to T cell exhaustion status in these contexts. Future research should prioritize understanding the differences in T cell exhaustion under different states (transplantation vs. tumor or infection), and the distinctions of exhaustion within T cell subsets themselves. Although non-memory T, B lymphocytes, and NK cells have a short life span and it is difficult to study their exhaustion phenomenon, their interaction with exhausted T cells is still worthy of attention. Elucidating the optimal levels of T cell exhaustion, for achieving long-term graft survival while maintaining protective immunity.

## Author contributions

XJW: Conceptualization, Writing – original draft, Writing – review & editing. JHZ: Data curation, Software, Visualization, Supervision, Writing – original draft, Writing – review & editing. PSZ: Formal analysis, Methodology, Writing – review & editing. XWW: Formal analysis, Supervision, Project administration, Writing – review & editing.
